# Synchronized Cell Cycle Arrest Promotes Osteoclast Differentiation

**DOI:** 10.3390/ijms17081292

**Published:** 2016-08-09

**Authors:** Minsuk Kwon, Jin-Man Kim, Kyunghee Lee, So-Young Park, Hyun-Sook Lim, Taesoo Kim, Daewon Jeong

**Affiliations:** 1 Laboratory of Bone Metabolism and Control, Department of Microbiology, Yeungnam University College of Medicine, Daegu 42415, Korea; kms1@boditech.co.kr (M.K.); kjinman75@hotmail.com (J.-M.K.); kyungheelee@ynu.ac.kr (K.L.); 2 Department of Physiology, Yeungnam University College of Medicine, Daegu 42415, Korea; sypark@med.yu.ac.kr; 3 Department of Public Health Administration, Hanyang Women’s University, Seoul 04763, Korea; limhs@hywoman.ac.kr; 4 Clinical Research Division, Korea Institute of Oriental Medicine, Daejeon 34054, Korea; xotn91@kiom.re.kr

**Keywords:** cell cycle arrest, cell synchronization, osteoclast differentiation

## Abstract

Osteoclast progenitors undergo cell cycle arrest before differentiation into osteoclasts, induced by exposure to macrophage colony-stimulating factor (M-CSF) and receptor activator of nuclear factor-κB ligand (RANKL). The role of such cell cycle arrest in osteoclast differentiation has remained unclear, however. We here examined the effect of synchronized cell cycle arrest on osteoclast formation. Osteoclast progenitors deprived of M-CSF in culture adopted a uniform morphology and exhibited cell cycle arrest at the G_0_–G_1_ phase in association with both down-regulation of cyclins A and D1 as well as up-regulation of the cyclin-dependent kinase inhibitor p27^Kip1^. Such M-CSF deprivation also promoted the differentiation of osteoclast progenitors into multinucleated osteoclasts expressing high levels of osteoclast marker proteins such as NFATc1, c-Fos, Atp6v0d2, cathepsin K, and integrin β3 on subsequent exposure to M-CSF and RANKL. Our results suggest that synchronized arrest and reprogramming of osteoclast progenitors renders them poised to respond to inducers of osteoclast formation. Further characterization of such effects may facilitate induction of the differentiation of heterogeneous and multipotent cells into desired cell lineages.

## 1. Introduction

Temporal coupling of cell cycle arrest and cell differentiation appears to be universal during organismal development [1]. Cell cycle arrest thus occurs prior to the differentiation of preadipocytes into adipocytes [2]. The transcription factor Prospero simultaneously regulates the expression of multiple cell cycle regulatory genes and neuronal lineage developmental genes in *Drosophila* [3]. The antiproliferative protein B cell translocation gene 1 (BTG1) is expressed at cell confluence as well as at the onset of myoblast differentiation, and its overexpression concurrently induces cell cycle arrest and terminal differentiation [4]. MyoD, a skeletal muscle-specific transcriptional regulator, coordinates skeletal muscle differentiation during cell cycle arrest in the G_0_–G_1_ phase by inducing the expression of the cyclin-dependent kinase (CDK)1 inhibitor p21 [5,6]. Additionally, forced silencing of proliferative signaling stimulates the differentiation of embryonic stem cells [7]. The precise nature of the relation between cell cycle arrest and the induction of differentiation has remained unclear, however.

Osteoclast differentiation in mammals is mediated by two osteoclastogenic factors: Macrophage colony-stimulating factor (M-CSF) and receptor activator of nuclear factor-κB ligand (RANKL), a member of the TNF family of proteins. Both *op*/*op* mutant mice (which are deficient in M-CSF) and RANKL-deficient mice manifest osteopetrotic bone defects as a result of the impaired formation of bone-resorptive osteoclasts [8,9]. M-CSF and RANKL play distinct roles in osteoclast formation by contributing to the regulation of osteoclast progenitor proliferation and the differentiation of these cells into multinucleated mature osteoclasts, respectively [8,9]. RANKL induces cell cycle arrest in G_0_–G_1_ in association with up-regulation of the CDK inhibitor p27^Kip1^ in a manner dependent on the interaction of RANKL with its cognate receptor RANK and the recruitment of TRAF6 (TNF receptor-associated factor 6) to the intracellular domain of RANK [10]. It has also been reported that RANKL-induced CDK6 down-regulation or RANKL-induced cell cycle arrest with both up-regulation of both p21^CIP1^ and p27^KIP1^ may be implicated in osteoclast differentiation [11,12]. Further, TNF-α—another osteoclastogenic factor—is known to induce G_1_ arrest in endothelial cells in association with the down-regulation of cyclin D1 and CDK2 and with up-regulation of the CDK inhibitors p16^INK4a^, p21^Waf^, and p27^Kip1^ [13].

To shed light on the role of cell cycle arrest during osteoclast differentiation, we have examined whether such arrest directly influences the differentiation process. We found that synchronized G_0_–G_1_ arrest induced by withdrawal of the proliferative factor M-CSF promotes osteoclast differentiation.

## 2. Results and Discussion

### 2.1. M-CSF Deprivation Induces G_0_–G_1_ Cell Cycle Arrest

**Figure 1:**
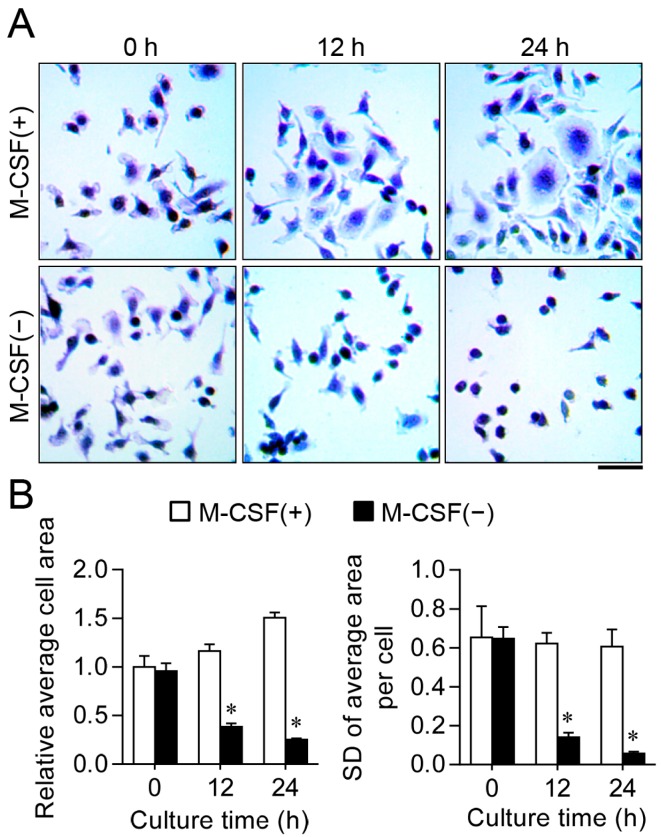
Effects of macrophage colony-stimulating factor (M-CSF) deprivation on the morphology and size of osteoclast progenitors. (**A**) Cells were cultured in the absence or presence of M-CSF for the indicated times and then stained with crystal violet. Scale bar: 50 µm; (**B**) Relative average cell surface area was determined by dividing the total cell area by the number of cells (**left panel**), and SD of the average area per cell was determined by measuring the area of individual cells (**right panel**), in photographs similar to those in (**A**). * Differences compared with control were statistically significant (*p* < 0.01, ANOVA).

To induce cell cycle synchronization, we cultured osteoclast progenitors in the absence of M-CSF for 12 h. Whereas cells cultured in the presence of M-CSF manifested a spindle and salverform morphology, those deprived of M-CSF for 12 or 24 h adopted a more spherical shape (Figure 1A). The surface area of the M-CSF-deprived cells decreased with time, in contrast with the increase apparent for cells cultured with M-CSF (Figure 1B). The uniformity of cell size was evaluated by calculation of the SD for the average area per cell, with a lower SD denoting a greater uniformity. The SD was markedly lower for cells cultured in the absence of M-CSF than for those maintained in its presence (Figure 1B). These results thus indicated that M-CSF-deprived cells were largely homogeneous in terms of cell morphology and size.

**Figure 2:**
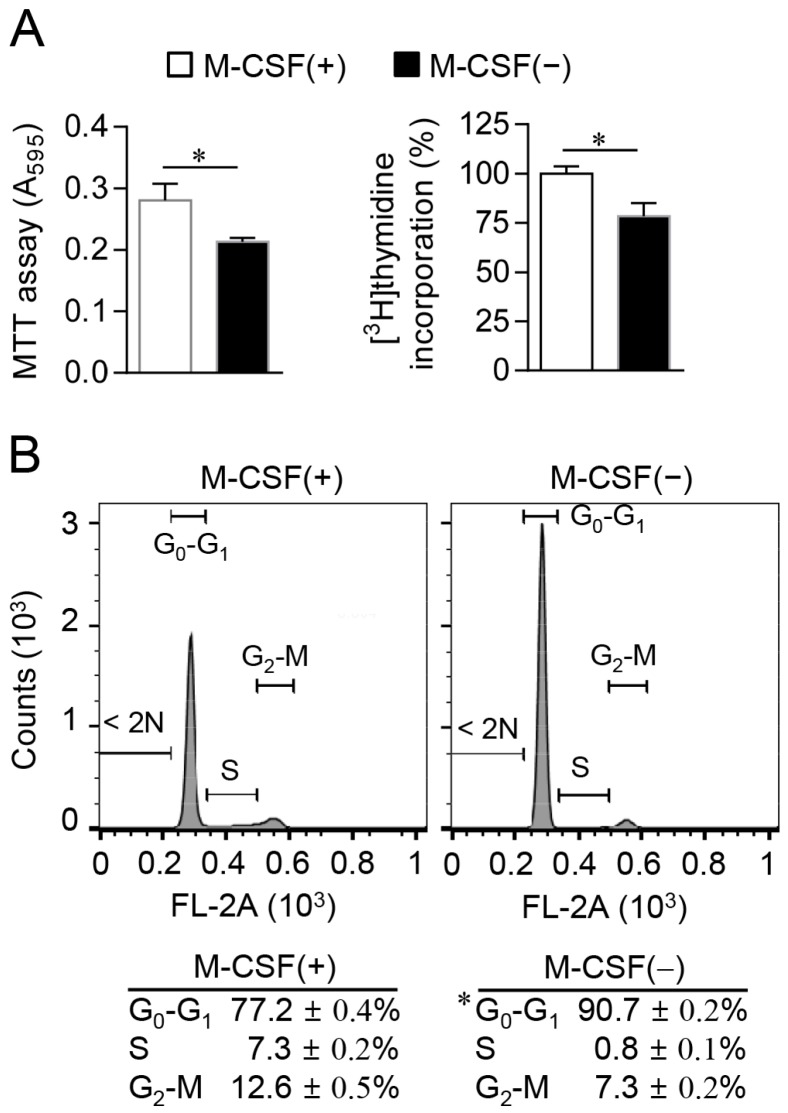
Induction of G_0_–G_1_ cell cycle arrest in osteoclast progenitors by M-CSF deprivation. (**A**) Cells were cultured in the absence or presence of M-CSF for 12 h, after which cell proliferation was determined with the MTT assay (**left panel**) or by measurement of [^3^H]thymidine incorporation (**right panel**); (**B**) Cells cultured as in (**A**) were stained with propidium iodide and subjected to cell cycle analysis by flow cytometry. Data are means ± SD for a representative experiment run in triplicate. * *p* < 0.01 (Student’s *t* test).

**Figure 3:**
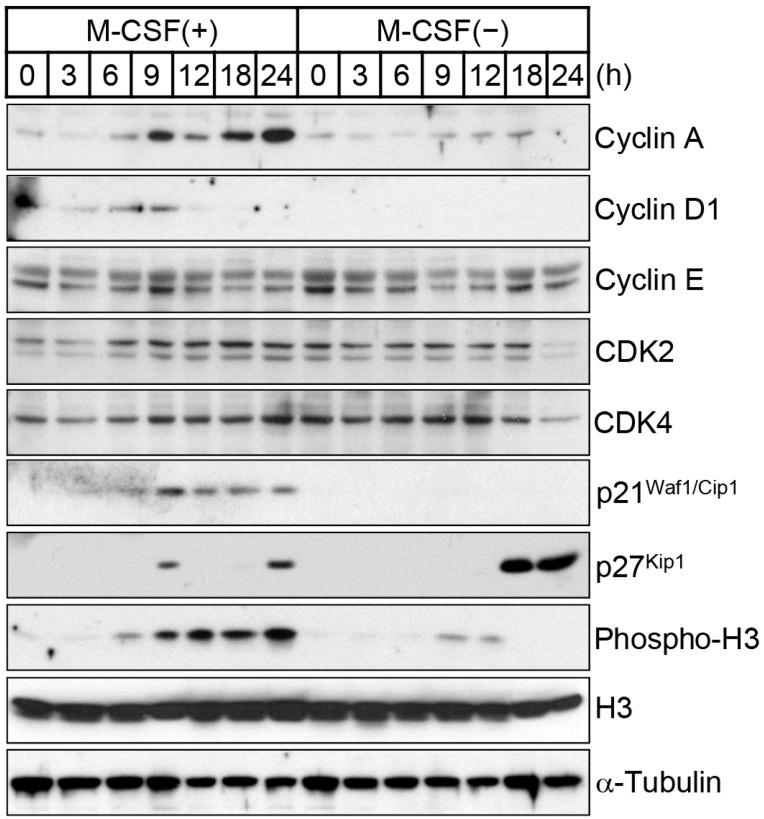
Change in expression levels of cell cycle regulators during M-CSF deprivation. Cells cultured for the indicated times were lysed and subjected to immunoblot analysis with antibodies to the indicated proteins.

We next measured the proliferation of osteoclast progenitors, both with the MTT assay and by measurement of [^3^H]thymidine incorporation into chromosomal DNA during the S phase of the cell cycle [14]. Both approaches confirmed that withdrawal of M-CSF for 12 h resulted in inhibition of cell proliferation (Figure 2A). Flow cytometric analysis of cells stained with propidium iodide also revealed that the proportion of cells in the S or G_2_-M phases of the cell cycle was reduced, whereas the proportion of those in the G_0_–G_1_ phase was increased in response to M-CSF deprivation (Figure 2B). Consistent with these results, immunoblot analysis of cell cycle regulators showed that the abundance of positive regulators of G_1_-phase CDKs—including cyclin A and cyclin D1—was reduced, whereas that of the G_1_-phase CDK inhibitor p27^Kip1^ was increased in cells deprived of M-CSF compared with those maintained in its presence (Figure 3). Furthermore, M-CSF withdrawal inhibited phosphorylation of histone H3 at Ser^10^, an event associated with S-phase entry [15]. The G_0_–G_1_ cell cycle arrest induced by M-CSF deprivation in osteoclast progenitors thus appeared to be due to down-regulation of cyclins A and D1 as well as up-regulation of the CDK inhibitor p27^Kip1^.

### 2.2. Cell Synchronization Promotes Osteoclast Formation

**Figure 4:**
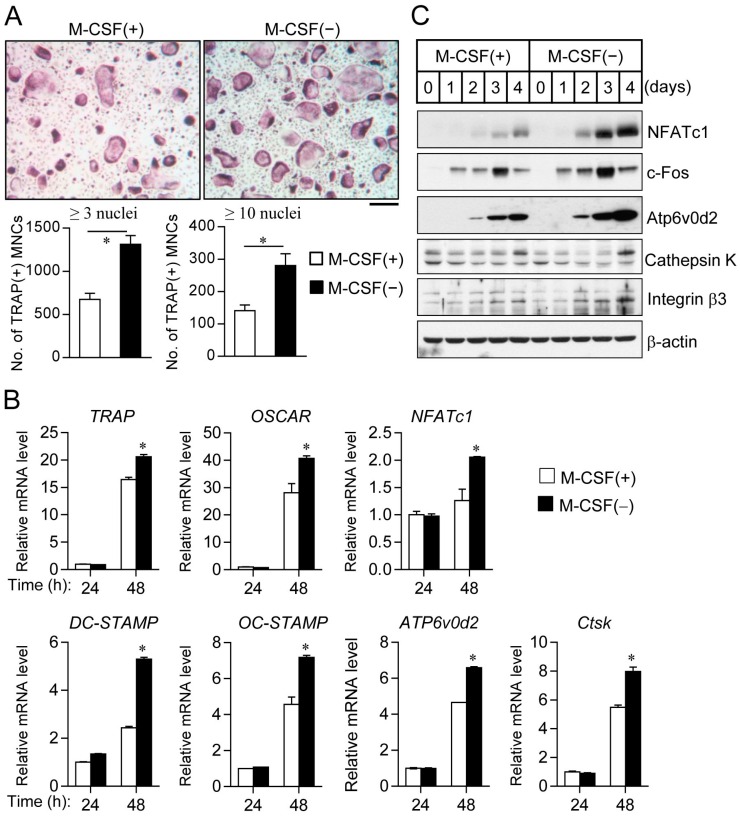
Prior M-CSF deprivation promotes osteoclast differentiation. (**A**) Osteoclast progenitors were cultured in the absence or presence of M-CSF for 12 h and were then exposed to M-CSF and receptor activator of nuclear factor-κB ligand (RANKL) for 4 days to induce osteoclast differentiation. The cells were then stained for tartrate-resistant acid phosphatase (TRAP, **upper panels**), and the number of TRAP(+) MNCs with ≥3 or ≥10 nuclei were counted (**lower panels**). Scale bar: 200 µm; (**B**) Osteoclast precursors with or without M-CSF were differentiated into osteoclasts for the indicated times. The mRNA levels of osteoclastogenic marker genes, including TRAP, OSCAR, NFATc1, DC-STAMP, OC-STAMP, ATP6v0d2, and cathepsin K (Ctsk). Quantitative data are means ± SD; * *p* < 0.01 (Student’s *t* test); (**C**) Immunoblot analysis of osteoclast marker proteins for osteoclast progenitors cultured in the absence or presence M-CSF for 12 h and then exposed to M-CSF and RANKL for the indicated times.

A molecular link between cell cycle withdrawal and cell differentiation has previously been suggested [7]. Furthermore, we found that osteoclast progenitors deprived of M-CSF appear to be homogeneous in terms of cell morphology and cell cycle progression. These observations together with others suggest a possible link between cell synchronization and osteoclast differentiation. Regarding the induction of osteoclast differentiation after culture in the absence of M-CSF, reprogrammed osteoclast progenitors manifested a marked increase in the formation of tartrate-resistant acid phosphatase-positive multinucleated cells (TRAP(+) MNCs) containing ≥3 or ≥10 nuclei compared with progenitors not previously deprived of M-CSF (Figure 4A). In addition, the expression of bone resorption-related proteases (MMP-9 and cathepsin K) in cell lysates and culture media was highly up-regulated in the bone-resorbing process of osteoclasts induced by M-CSF withdrawal than in the control (Figure S1). However, the osteoclastic cell area of TRAP(+) MNCs containing ≥3 nuclei was not different between control and M-CSF-withdrawal cells. Additionally, bone resorption pit formation of mature osteoclasts and the content of bone-resorptive end product from type I collagen (deoxypyridinoline, DPD) showed no significant difference between control and M-CSF-deprived cells, due to the survival and longevity of osteoclasts with a full actin ring during bone resorption (Figures S2 and S3). We also observed that the differentiation of synchronized cells upon cell–cell contact inhibition or serum withdrawal showed a similar result as compared to cells synchronized by M-CSF deprivation alone (Figure S4). Further, enhanced osteoclast differentiation by prior M-CSF withdrawal was confirmed by real-time PCR (Figure 4B) or immunoblot analysis (Figure 4C), showing increased expression of various osteoclastic markers, including TRAP, osteoclast-associated immunoglobulin-like receptor (OSCAR), the osteoclastogenic transcription factors NFATc1 (nuclear factor of activated T cells c1) and c-Fos (component of AP-1), as well as dendrocyte-expressed seven transmembrane protein (DC-STAMP), osteoclast stimulatory transmembrane protein (OC-STAMP), and Atp6v0d2 (fusion factor of mononuclear osteoclast precursors), cathepsin K (bone-resorptive cysteine protease), and integrin β3 chain (subunit of integrin αvβ3).

Our findings show that M-CSF deprivation induces cell cycle arrest at the G_0_–G_1_ phase, elicits the adoption of a uniform cell morphology, and promotes the subsequent induction of osteoclast formation in osteoclast progenitors. This concept would be especially important in the differentiation of synchronized osteoclast progenitors into dendritic cells in the future. In addition to its induction of cell cycle arrest, deprivation of M-CSF may silence intracellular signaling networks and thereby increase cell sensitivity to new extracellular cues, rendering osteoclast progenitors poised to respond to the induction of osteoclast differentiation by RANKL. More generally, withdrawal of nutrients (such as glucose and amino acids), growth factors, or other receptor ligands may serve to reprogram cells to confer enhanced susceptibility to inducers of differentiation. Further studies are warranted to determine the effects of such cell synchronization on differentiation efficiency in multipotent stem cells, cancer cells, graft cells, and tissue resident cells. Such knowledge may serve to facilitate the induction of the differentiation of multipotent or heterogeneous cells into specific cell types of interest.

## 3. Materials and Methods

### 3.1. Induction of Synchronized Cell Cycle Arrest and Osteoclast Differentiation

Mononuclear osteoclast progenitors were isolated from bone marrow of mice as described previously [16], and were cultured in α-minimum essential medium (α-MEM; Invitrogen, Carlsbad, CA, USA) supplemented with antibiotics, 10% FBS, and recombinant human M-CSF (30 ng/mL). Osteoclast progenitors at 70% confluence (5 × 10^4^ cells per well in 48-well culture plates) were induced to undergo cell cycle arrest by exposure to culture medium lacking M-CSF for 12 h. For induction of osteoclast differentiation, the M-CSF-deprived or control cells were cultured in medium containing M-CSF (30 ng/mL) and recombinant mouse RANKL (100 ng/mL) for 4 days, with replenishment of the medium after 2 days. Osteoclast differentiation was assessed by staining of the cells for tartrate-resistant acid phosphatase (TRAP) with the use of a leukocyte acid phosphatase staining kit (Sigma-Aldrich, St. Louis, MO, USA). TRAP-positive multinucleated cells (TRAP(+) MNCs) containing ≥3 or ≥10 nuclei were counted under a light microscope.

### 3.2. Analysis of Cell Area

Osteoclast progenitors (5 × 10^4^ cells per well in 48-well culture plates) were incubated in culture medium with or without M-CSF (30 ng/mL) for the indicated times. The cells were then fixed with 3.7% formalin for 10 min, stained with 0.5% crystal violet for 30 min, washed with PBS, and photographed under a light microscope for measurement of cell surface area with the use of Image-Pro plus software version 6.0 (Media Cybernetics, Silver Spring, MD, USA).

### 3.3. Assay of Cell Proliferation and Cell Cycle Analysis

For assay of cell proliferation, osteoclast progenitors were incubated in culture medium with or without M-CSF (30 ng/mL) for 12 h before exposure to 3-(4,5-dimethylthiazol-2-yl)-2,5-diphenyltetrazolium bromide (MTT). The formazan product was then dissolved in DMSO and quantitated spectrophotometrically at a wavelength of 595 nm. Alternatively, cells (1 × 10^5^ cells per well in 24-well culture plates) were incubated in culture medium containing [^3^H]thymidine (1 μCi per well; PerkinElmer, Waltham, MA, USA) in the absence or presence of M-CSF for 12 h, washed with ice-cold PBS, treated with ice-cold 5% trichloroacetic acid (TCA), and washed again with ice-cold PBS before preparation of cell lysates with a lysis solution containing 0.5% SDS and 0.5 M NaOH. The lysates were mixed with liquid scintillation cocktail (DCC-BIONET, Seongnam, Korea), and the amount of [^3^H]thymidine-labeled DNA was measured with a liquid scintillation counter (Tri-Carb 3110 TR, PerkinElmer, Santa Clara, CA, USA).

For cell cycle analysis, osteoclast progenitors were incubated in culture medium with or without M-CSF (30 ng/mL) for 12 h and were then detached from the plate by exposure to trypsin and isolated by centrifugation at 1000× *g* for 5 min. The cells were suspended in PBS containing 5 mM EDTA, fixed with 70% ethanol for 12 h at 4 °C, isolated again by centrifugation, resuspended in PBS containing 5 mM EDTA, treated with RNase A (50 μg/mL) for 30 min at room temperature, and stained with propidium iodide (50 μg/mL) for 10 min in the dark. The stained cells were analyzed by flow cytometry with a FACSCalibur instrument (Becton Dickinson, San Jose, CA, USA).

### 3.4. Immunoblot Analysis

For analysis of cell cycle regulators or osteoclast marker proteins, cells incubated in culture medium with or without M-CSF (30 ng/mL) and then exposed (or not) to M-CSF and RANKL for the indicated times were lysed and subjected to immunoblot analysis with antibodies to cyclin A, to cyclin D1, to cyclin E, to CDK2, to CDK4, to p21^Waf1/Cip1^, to p27^Kip1^, to Ser^10^-phosphorylated histone H3, to α-tubulin, to NFATc1, to Atp6v0d2, to integrin β3, and to β-actin (all from Santa Cruz Biotechnology, Santa Cruz, CA, USA); with those to histone H3 and to cathepsin K (Abcam, Cambridge, MA, USA); and with those to c-Fos (Cell Signaling Technology, Boston, MA, USA).

### 3.5. Statistical Analysis

Quantitative data are presented as means ± SD from at least three independent experiments. Differences between two groups were analyzed with Student’s *t* test. For statistical analysis for multiple comparisons, means between multiple groups were performed using one-way ANOVA analysis using the Microsoft 2010 Excel program. A *p* value of <0.05 was considered statistically significant.

## Supplementary Materials

Supplementary materials can be found at www.mdpi.com/1422-0067/17/8/1292/s1.
