# Supplementary material for: Synchronized Cell Cycle Arrest Promotes Osteoclast Differentiation
Source: Int J Mol Sci. 2016 Aug 9;17(8):1292. doi: 10.3390/ijms17081292 (PMC5000689; doi:10.3390/ijms17081292)
Supplement: Supplementary file 1 [file ijms-17-01292-s001.pdf]

Supplementary Materials: Synchronized Cell Cycle Arrest Promotes Osteoclast Differentiation

Minsuk Kwon, Jin-Man Kim, Kyunghee Lee, So-Young Park, Hyun-Sook Lim, Taesoo Kim and Daewon Jeong

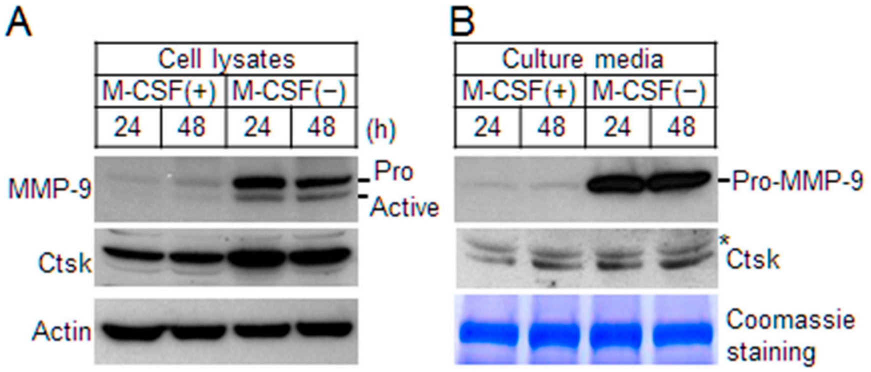

Figure S1. The expression level of bone-resorptive proteases during pit formation. Osteoclast precursors with or without M-CSF withdrawal for 12 h were differentiated into osteoclasts in the presence of M-CSF and RANKL for 4 days, after which osteoclasts were further incubated for the indicated times, and cell lysates (**A**) and culture media (**B**) were subjected to immunoblotting with specific antibodies to matrix metalloproteinase-9 (MMP-9) and cathepsin K (Ctsk). The actin and coomassie-stained band were used a loading control. *: non-specific band.

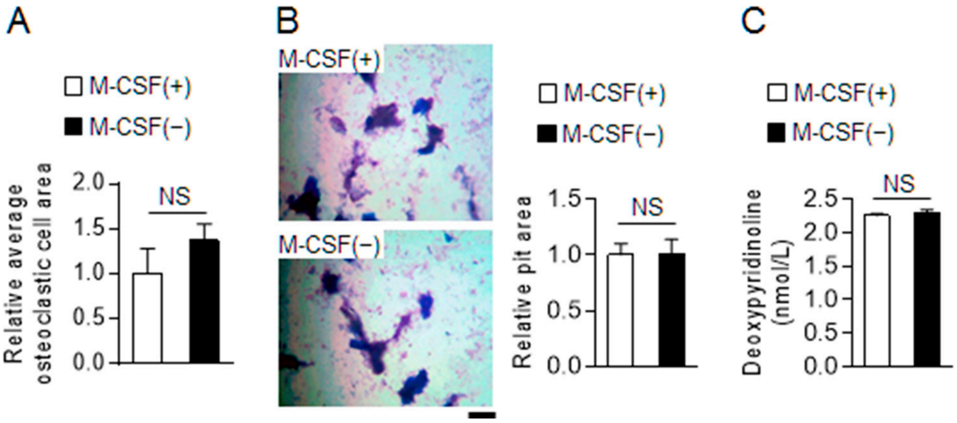

Figure S2. Change of osteoclastic cell size in M-CSF-deprived cells and bone pit formation assay. (**A**) Osteoclast progenitors were cultured in the absence or presence of M-CSF for 12 h. After exposure to M-CSF and RANKL for 4 days to induce osteoclast differentiation, the cells were stained for TRAP and relative average osteoclastic cell area of TRAP(+) MNCs with ≥3 nuclei was determined by dividing the total cell area by the number of cells; (**B**) Osteoclast progenitors were seeded on dentine slices (IDS Ltd., Boldon, UK) in the absence or presence of M-CSF for 12 h and differentiated into mature osteoclasts in the presence of M-CSF and RANKL for 4 days followed by a further incubation for 2 days to resorb bone. The slices were stained with 1% toluidine blue and the resorption pit area was analyzed using Image-Pro Plus version 6.0 software (MediaCybernetics, Silver Spring, MD, USA). Scale bar: 50 µm; (**C**) Bone-resorptive end product, deoxypyridinoline (DPD), in culture media as in (**B**) was determined using MicroVue DPD EIA kit (Quidel, San Diego, CA, USA). NS stands for Not Significant.

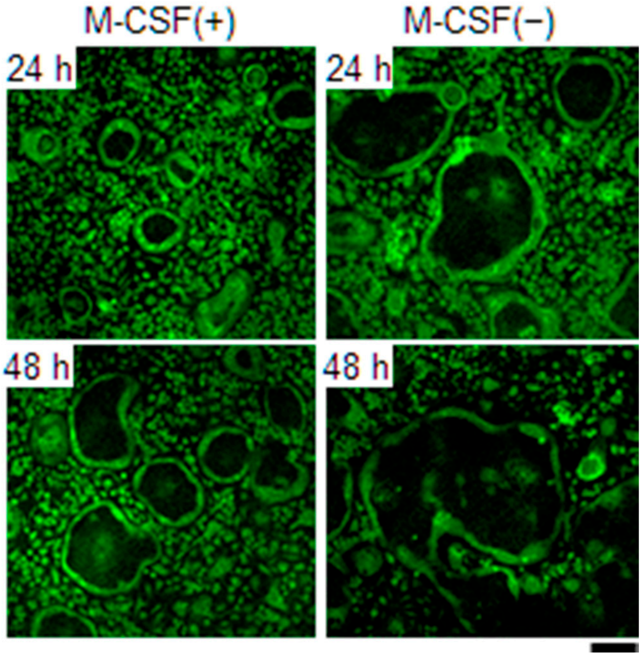

Figure S3. F-actin staining in osteoclasts. Osteoclast precursors with or without M-CSF withdrawal for 12 h were differentiated into osteoclasts in the presence of M-CSF and RANKL for 4 days, after which osteoclasts were further cultured for the indicated times, fixed, permeabilized, stained with fluorescein isothiocyanate (FITC)-conjugated phalloidin (Invitrogen, Carlsbad, CA, USA), and examined by fluorescence microscopy. Scale bar: 100 μm.

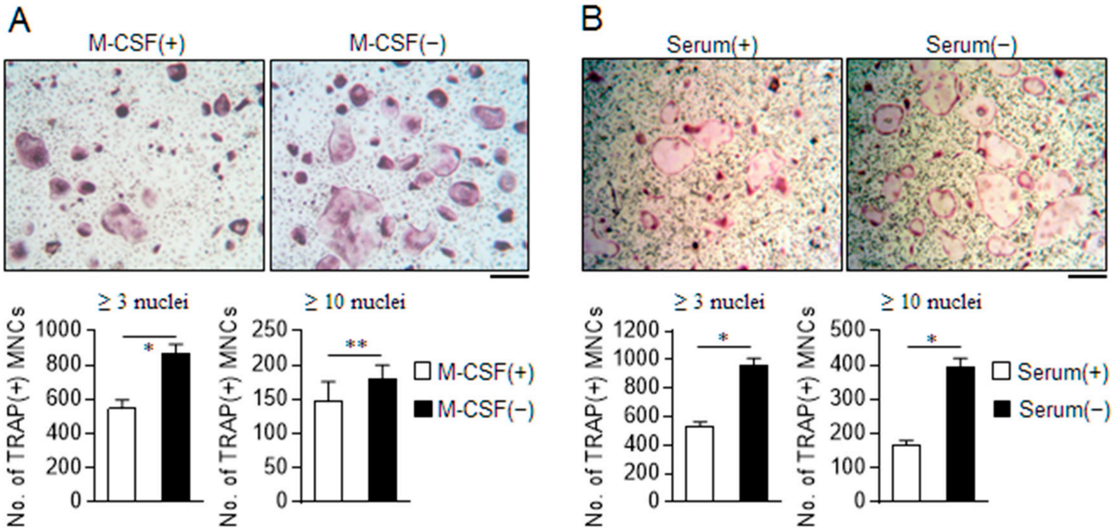

Figure S4. Increased osteoclast differentiation by cell-cell contact inhibition or serum withdrawal. (**A**) Osteoclast progenitors (2×10^5 cells per well) were seeded in 48-well plates to reach 100% confluence and cultured in the absence or presence of M-CSF for 12 h, after which osteoclast differentiation was achieved by treating with M-CSF and RANKL for 4 days; (**B**) Osteoclast progenitors (5×10^4 cells per well in 48-well culture plates) were incubated in culture medium with M-CSF plus 10% FBS [Serum (+)] or 0.5% FBS only [Serum (−)] for 12 h and further differentiated into osteoclasts in the presence of M-CSF and RANKL for 4 days. After cells as in (**A**) and (**B**) were stained for TRAP, the number of TRAP(+) MNCs with ≥3 or ≥10 nuclei were counted under a light microscope. Scale bar: 200 μm. Data are means ± SD * $p < 0.01$, ** $p < 0.05$ (Student's *t* test).
